# Acid Mine Drainage Treatment Using Bayer Precipitates Obtained from Seawater Neutralization of Bayer Liquor

**DOI:** 10.1002/gch2.201800061

**Published:** 2018-09-21

**Authors:** Gurkiran Kaur, Sara J. Couperthwaite, Graeme J. Millar

**Affiliations:** ^1^ Chemistry, Physics, Mechanical Engineering, Science and Engineering Faculty Queensland University of Technology (QUT) Brisbane 4001 Queensland Australia; ^2^ Institute for Future Environments Queensland University of Technology (QUT) Brisbane 4001 Queensland Australia

**Keywords:** acid mine drainage, bauxite refinery residues, Bayer precipitates, seawater neutralization

## Abstract

Bayer precipitates from the seawater neutralization of Bayer liquor waste from the alumina industry are shown to be a prospective solution for the remediation of acid mine drainage (AMD) water. Precipitates are varied in composition, albeit they are generally comprised of hydrotalcite (Mg_6_Al_2_(OH)_16_CO_3_∙*x*H_2_O), calcite (CaCO_3_), aragonite (CaCO_3_), mixed metal hydroxides (Mg_2_Al(OH)_7_), and halite (NaCl). Brucite (Mg(OH)_2_) is detected for lower Bayer liquor concentrations (1–3 g L^−1^ Al_2_O_3_) when the concentrations of aluminum and hydroxyl species are insufficient to promote hydrotalcite formation. The neutralizing capacity of the precipitates also varies with Bayer liquor composition. Treatment of AMD water with Bayer precipitates met discharge pH guidelines. The dissolution of hydrotalcite and brucite (1–3 g L^−1^ Bayer precipitates only) is responsible for the Bayer precipitate's neutralizing capacity, while calcium carbonate has a buffering affect at around pH 7. Manganese ions are the most challenging species to remove because high pH values are required (pH > 9), which is not possible with all precipitates tested. One caveat is that increasing the degree of manganese removal generates issues with excessive dissolved aluminum which exceeds discharge limits. Future research should address this latter problem and facilitate implementation of this approach to AMD remediation.

## 1 Introduction

The Bayer process developed and patented by Karl Joseph Bayer 110 years ago is used for refining bauxite ore into smelting grade alumina (Al_2_O_3_). The Bayer process involves the digestion of bauxite in concentrated caustic at high temperatures followed by precipitation, clarification, and calcination to recover the alumina content.^1^, ^2^, ^3^ The remaining insoluble solids from the extraction step of the Bayer process are known as bauxite refinery residues, which are composed of ≈45% liquor (Bayer liquor) and 55% solid (red mud).^3^, ^4^ The exact composition of the bauxite refinery residue depends on the initial type of bauxite ore and the digestion conditions (temperature and pressure) used at the refinery.^5^, ^6^ The obtained liquor is strongly alkaline (pH ranging from 10 to 13) due to the presence of high concentrations of sodium aluminate, sodium carbonate, and sodium hydroxide. Therefore, it is necessary to reduce the pH of the Bayer liquor to a value of 8.5–8.9 prior to disposal.^3^, ^7^

Bauxite refinery residues have been neutralized by various methods including infiltration of rainwater and atmospheric CO_2_, treatment with strong acids, gypsum addition, and seawater neutralization.^8^, ^9^, ^10^ Alumina refineries located in coastal areas in particular have implemented the neutralization of the bauxite refinery residue with seawater, a process which provides a reduction in both pH and dissolved metal concentrations.^11^, ^12^ Seawater neutralization of bauxite refinery residue results in the reduction of alkalinity through the precipitation of Mg, Ca, and Al hydroxides and carbonate minerals.^10^, ^11^, ^13^, ^14^ The primary pathway of the seawater neutralization process is the formation of Bayer hydrotalcite, a stable alkaline precipitate consisting of positively charged metal hydroxide layers (Mg and Al) which are balanced through the intercalation of anions (CO_3_^2−^ in Bayer liquor) into the interlayers. The formation of Bayer hydrotalcite has been proposed to occur as represented in Equation (1), 15

(1) $$\begin{array}{l} 6{\mathrm{MgCl}}_{2(\mathrm{aq})}+2\mathrm{NaAl}{\left(\mathrm{OH}\right)}_{4(\mathrm{aq})}+8{\mathrm{NaOH}}_{\left(\mathrm{aq}\right)}\\ \;\;\;+{\mathrm{Na}}_{2}{\mathrm{CO}}_{3(\mathrm{aq})}\to {\mathrm{Mg}}_{6}{\mathrm{Al}}_{2}{\left(\mathrm{OH}\right)}_{16}\left({\mathrm{CO}}_{3}\right)\cdot xH_{2}O_{\left(s\right)}+12{\mathrm{NaCl}}_{\left(s\right)} \end{array}$$

Impoundment‐type bauxite residue storage facilities consist of solid bauxite residue (red mud) and an alkaline supernatant liquor (SNL); entrained liquor in bauxite residue and leachates from the residue with rainwater.^16^ The ability of red mud to neutralize acidic water and to remove heavy metals from mine sites has been studied by several researchers.^13^, ^17^, ^18^, ^19^, ^20^, ^21^, ^22^ Couperthwaite et al.^23^ reported that due to the formation of Bayer hydrotalcite, seawater neutralized red mud exhibited greater potential to treat acid sulfate soils compared to untreated red mud.

SNL typically has a relatively low alumina content and alkalinity compared to superevaporative liquor (after digestion) and underflow residue washer liquor,^24^ as well as a variable composition of anions and heavy metals at trace but potentially harmful concentrations to the environment.^16^ The composition of SNL is highly dependent on the processing conditions (bauxite ore, digestion, liquor purification), disposal method (lagooning and dry stacking, and whether there was prior neutralization before disposal), and climatic factors (rainfall and evaporation rates).^16^ Impoundment of SNL is essential to minimize the risk of surface and groundwater contamination.^16^, ^24^ Storage of SNL causes increased storage capacity requirements for bauxite refinery storage facilities, and thus methods such as seawater neutralization have been trialed as a means of reducing SNL volumes.^16^ Seawater neutralization reacts SNL from impoundments with evaporative seawater (heated to 50 °C), which is then discharged through a labyrinth of settlement channels to produce an inert precipitate and water suitable for estuary discharge.^16^ Notably, the latter precipitate (calcium carbonate and hydrotalcite) still requires a suitable disposal technique or more preferably a beneficial reuse option.

As the precipitate formed from seawater neutralized supernatant liquor is alkaline, a possible route for reuse is the remediation of acidic waste solutions. The mining industry has generated literally thousands of sites which are afflicted with problems associated with acid mine drainage (AMD).^25^ AMD is characterized by high sulfate and metal content and is created when sulfide minerals in the waste rock and tailings are exposed to the atmosphere; thus, oxidizing to form sulfuric acid, which then has the ability to leach heavy metals from surrounding rock.^26^, ^27^ A major concern with AMD solutions is run‐off into neighboring waterways which may represent an environmental problem, hence there is a demand to develop effective remediation methods.^28^, ^29^ Therefore, the application of seawater neutralized supernatant liquor to neutralize AMD may be commercially and socially attractive.

However, the variability in composition of bauxite residues due to different ore compositions and process conditions may result in variable neutralization capacity.^30^, ^31^, ^32^, ^33^ Consequently, there is a need to understand the reaction conditions that influence the formation of Bayer precipitates to identify synthesis conditions that result in optimal materials for the treatment of acid mine drainage or acid sulfate soils. The hypothesis was that relative availability of reactive species in the Bayer liquor (aluminate, hydroxide, and carbonate) and seawater (magnesium and calcium concentrations) can affect the formation of precipitates, particularly Bayer hydrotalcite. Therefore, this investigation addressed the following research issues: (1) How does the Bayer liquor constitution influence the composition, structure, and stability of the obtained precipitates upon seawater addition? (2) What is the impact of Bayer liquor composition upon neutralization efficiency of seawater? and (3) How effective is the treatment of AMD using precipitates from neutralization of Bayer liquor? Bayer precipitates were synthesized using a batch process that simulated the seawater neutralization process used in industry, and these materials were then characterized by X‐ray diffraction (XRD), infrared spectroscopy (IR), ion coupled plasma‐optical emission spectroscopy (ICP‐OES), and thermogravimetric analysis (TGA).

## 2 Results and Discussion

### 2.1 Impact of Bayer Liquor Composition on Seawater Neutralization Precipitates

**Figure 1:**
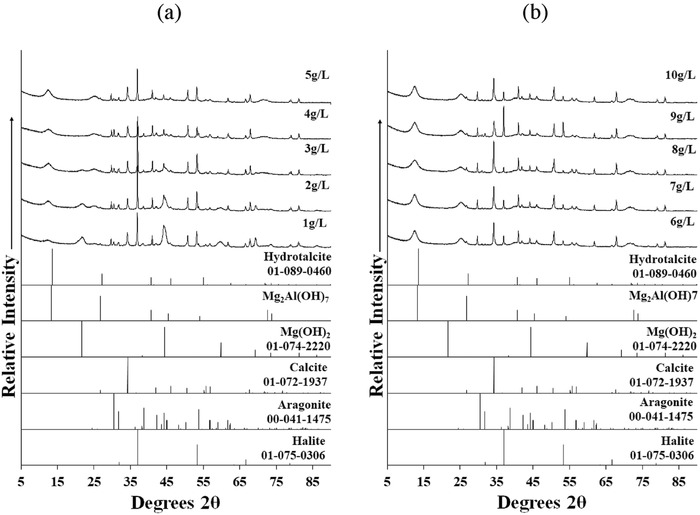
XRD patterns for Bayer precipitates formed at pH 9.25: a) 1–5 g L^−1^ Al_2_O_3_ and b) 6–10 g L^−1^ Al_2_O_3_.

To illustrate the variations in the precipitates formed by seawater addition, characterization of materials formed at pH 9.25 with Bayer liquor at 1–10 g L^−1^ Al_2_O_3_ concentration was conducted. A final pH of 9.25 was chosen on the basis that this pH satisfied regulations for safe disposal of supernatant.^15^, ^34^ **Figure** **1** shows XRD patterns of Bayer precipitates formed at a pH value of 9.25.

There was evidence for the formation of hydrotalcite (Mg_6_Al_2_(OH)_16_CO_3_), mixed metal hydroxides (Mg_2_Al(OH)_7_), calcite (CaCO_3_), aragonite (CaCO_3_), and halite (NaCl) in all obtained precipitates based on matches from respective reference patterns. Several studies regarding the seawater neutralization of bauxite refinery residues report similar mineralogical phases.^12^, ^35^, ^36^ Greater insight regarding the precipitates formed is revealed in **Table** **1**, which summarized the results of quantitative XRD analysis of the Bayer precipitates.

**Table 1:** Phase composition of Bayer precipitates formed from seawater neutralization of 1–10 g L^−1^ Bayer liquor

Bayer precipitates	Relative percentage				
	Aragonite	Calcite	Halite	Brucite	Amorphous + layered double hydroxide (LDH)
1	4.6	8.4	8.4	52.9	25.7
2	5.7	5.6	9.0	30.0	49.8
3	5.8	6.4	9.7	13.4	64.7
4	11.1	8.4	5.3	–	75.1
5	2.3	9.0	7.4	–	81.4
6	0.3	10.7	6.4	–	82.6
7	0.2	12.2	1.8	–	85.8
8	0.9	13.5	0.6	–	85.0
9	0.2	13.3	2.3	–	84.2
10	0.2	13.3	0.9	–	85.6

It was noted that an additional brucite (Mg(OH)_2_) phase (reflections observed at ≈22° and 45° 2θ) was evident in 1–3 g L^−1^ Bayer precipitates, but absent in XRD spectra of 4–10 g L^−1^ Bayer precipitates (Figure 1). Brucite forms from the reaction of dissolved magnesium ions with hydroxide species according to Equation (2)

(2) $${\mathrm{Mg}}_{\left(\mathrm{aq}\right)}^{2+}+2{\mathrm{OH}}_{\left(\mathrm{aq}\right)}^{-}\to \mathrm{Mg}{\left(\mathrm{OH}\right)}_{2\left(s\right)}$$

A plausible explanation was the fact that the additional aluminum and hydroxide ions present in the 4–10 g L^−1^ Bayer liquor (**Table** **2**) facilitated the formation of the more thermodynamically stable hydrotalcite structure (Equation (1)). The XRD reflections ascribed to the presence of hydrotalcite did not fit exactly with the reference pattern from the database, which was consistent with the likely formation of several hydrotalcite species (range of Mg:Al: 2:1 at pH > 12, 3:1 at pH ≤ 10, and 4:1 at pH ≤ 8) during neutralization process.^11^ In addition, hydrotalcite‐related peaks appeared broad and of relatively low intensity, indicating that relatively small crystals formed.^15^, ^36^

**Table 2:** Composition of Bayer liquor and seawater

Bayer liquor concentration [g L^−1^ Al_2_O_3_]	pH	Concentration [mg L^−1^]					
		Al	Mg	S	Na	K	Ca
1	13.27	450.5	0.775	3.47	20 730	30.92	<0.05
2	13.35	1190	1.40	0.79	22 240	78.42	<0.05
3	13.37	1503	1.28	3.65	19 570	84.29	<0.05
4	13.40	2045	1.23	5.39	20 090	111.4	<0.05
5	13.45	2525	0.791	11.13	20 840	123.3	<0.05
6	13.48	3125	1.27	4.95	21 590	165.7	<0.05
7	13.51	3613	1.21	8.42	22 080	191.8	<0.05
8	13.53	4232	1.33	8.67	22 730	209.3	<0.05
9	13.55	4466	1.33	10.5	23 260	237.7	<0.05
10	13.61	4893	1.10	20.14	32 030	246.8	<0.05
Seawater	7.73	<0.05	1256	806.9	9429	628	395

Due to the presence of calcium in seawater, XRD patterns for all obtained Bayer precipitates show calcite and aragonite were formed along with hydrotalcite. Taylor et al.^7^ reported that in Bayer liquor, the main alkalinity providing species were hydroxides (OH^−^), aluminate ions (Al(OH)_4_^−^), and carbonates (CO_3_^2−^). Thus, the presence of calcium carbonate upon the addition of seawater (source of Ca) to Bayer liquor can be explained in terms of Equation (3)

(3) $${\mathrm{Ca}}_{\left(\mathrm{aq}\right)}^{2+}+{\mathrm{CO}}_{3\left(\mathrm{aq}\right)}^{2-}\to {\mathrm{CaCO}}_{3\left(s\right)}$$

An interesting trend existed for calcite and aragonite in the XRD patterns (Table 1), whereby calcite peaks were prevalent in the precipitates obtained from higher Bayer liquor concentrations (6–10 g L^−1^), while both aragonite and calcite related peaks were present in precipitates obtained at lower concentrations. It has been suggested that magnesium plays an important role in the precipitation of calcium carbonate polymorphs.^37^, ^38^ Calcite and aragonite formation depended upon the Mg:Ca ratio in solution,^39^ with relatively high magnesium in solution inhibiting the growth of calcite relative to aragonite. This latter conclusion was in harmony with this study, wherein it was found that for higher Bayer liquor concentrations the formation of hydrotalcite (Equation (1)) resulted in a low Mg:Ca ratio favoring calcite formation (Table 1).

The detection of amorphous phases by XRD may be consistent with the presence of Al(OH)_3_ species (Equation (4)) as aluminates were known to be present in Bayer liquor^7^

(4) $$\mathrm{Al}{\left(\mathrm{OH}\right)}_{4\left(\mathrm{aq}\right)}^{-}\;\to \mathrm{Al}{\left(\mathrm{OH}\right)}_{3\left(s\right)}+{\mathrm{OH}}_{\left(\mathrm{aq}\right)}^{-}$$

**Figure 2:**
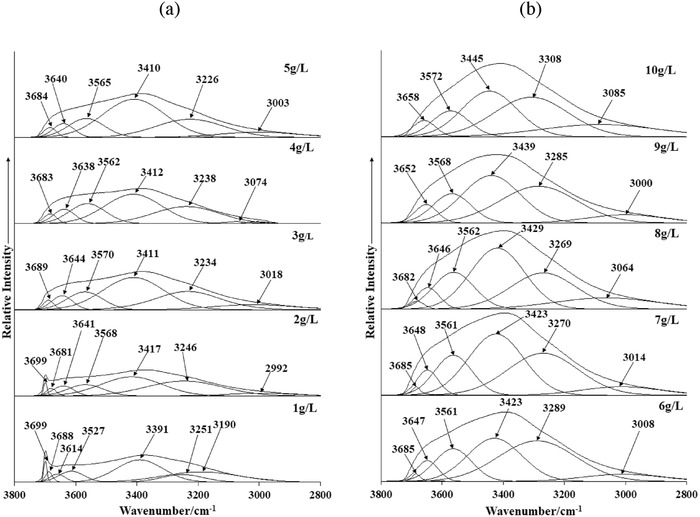
Infrared spectra (3800–2800 cm^−1^) for a) 1–5 g L^−1^ and b) 6–10 g L^−1^ Al_2_O_3_ samples.

**Figure 3:**
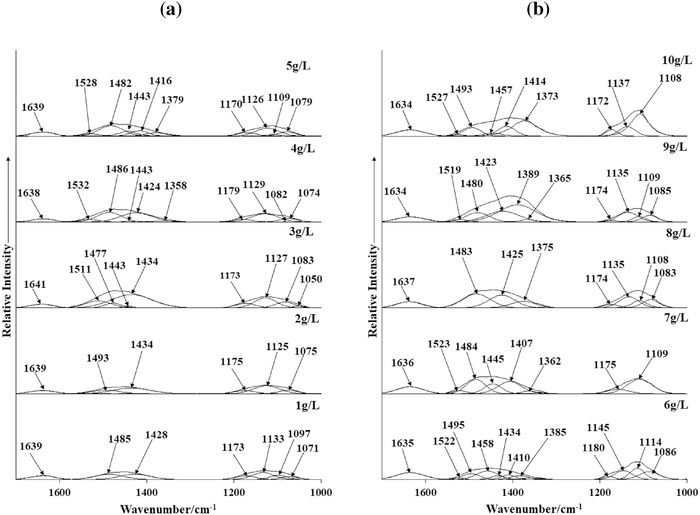
Infrared spectra (1650–1000 cm^−1^) for a) 1–5 g L^−1^ and b) 6–10 g L^−1^ Al_2_O_3_ samples.

Infrared spectroscopy was also conducted to verify and enhance the findings from XRD analysis of the Bayer precipitates (**Figures** **2** and **3**). In general, the infrared spectra for Bayer precipitates (1–10 g L^−1^) were comparable to peaks ascribed to synthetic hydrotalcite,^40^ and summarized in Table S1 (Supporting Information).^41^, ^42^

For all the obtained precipitates, a broad profile centered at 3400 cm^−1^ was observed, which was mainly due to a combination of the stretching modes of hydroxyl layers in the LDH structure and water molecules (Figure 2). Peak fitting revealed that a sharp peak at 3700–3690 cm^−1^ was present for the 1 and 2 g L^−1^ precipitates, ascribed to Mg—OH vibrations in brucite.^15^, ^43^, ^44^ This latter observation was consistent with the corresponding XRD patterns (Figure 1), which also indicated the presence of magnesium hydroxide under the same conditions. In terms of the remaining sub‐bands identified in the region 3690–3500 cm^−1^, these were assigned to vibrations of Mg—Al—OH bonds in hydrotalcite or in Mg_2_Al(OH)_7_.^45^

As the concentration of aluminum and hydroxide increased in solution, the formation of hydrotalcite was thermodynamically more stable.^46^ Alternatively, bands in the region 3500–3300 cm^−1^ were attributed to a number of overlapping OH‐stretching vibrations of water, presumably originating from the metal hydroxyl layers, intercalated water, and solvated anions.^47^ Peaks in the lower hydroxyl region 3200–2800 cm^−1^ were attributed to hydrogen bonding between water molecules and interlayer water.^41^, ^45^ For the 4–10 g L^−1^ Bayer precipitate samples, a general shift of the peak positions to higher wavenumbers in the region 3500–3300 cm^−1^ was observed. The intensity of the general OH/water vibrational profile additionally intensified with increasing Bayer liquor concentration, which suggested that more water was intercalated either as “free” water and/or as solvated intercalated anions.

The water deformation modes observed in the region 1660–1580 cm^−1^ provided information about the interlayer anions of hydrotalcites (e.g., bands at 1655 and 1631 cm^−1^ indicated the presence of sulfate and carbonate as interlayer anions, respectively).^11^ For all obtained precipitates, the water deformation band was observed at around 1635 cm^−1^, thus suggesting that the interlayer anion remained constant. The presence of a band between 1400 and 1350 cm^−1^ suggested carbonate as the dominant interlayer anion in hydrotalcite.^41^ The IR spectra of the carbonate antisymmetric stretching region (1500–1350 cm^−1^) displayed multiple bands (Figure 3), which were attributed to calcite (1460–1400 cm^−1^), aragonite (1500–1460 cm^−1^), and carbonates in the hydrotalcite interlayer (1400–1350 cm^−1^).^42^ Precipitates obtained for high Bayer liquor concentrations (6–10 g L^−1^) showed a sharp band in the region 1400–1350 cm^−1^ assigned to intercalated carbonate anions in the hydrotalcite structure. The relative intensity of the 1400–1350 cm^−1^ band increased as the Bayer liquor concentration increased, which suggested a possible increase in the number of carbonate anions in the interlayer region. The most intense carbonate band was observed in the region 1460–1400 cm^−1^ (characteristic of calcite)^35^, ^48^ for 6–10 g L^−1^ Bayer precipitates, while the band at ≈1500–1460 cm^−1^ (characteristic of aragonite)^34^, ^48^ was present for 1–5 g L^−1^ Bayer precipitates in addition to calcite bands. These latter trends supported XRD results that calcite formation was favored for precipitates formed at 6–10 g L^−1^ Bayer liquor concentrations, while for 1–5 g L^−1^ Bayer liquor concentrations both aragonite and calcite formed.

The precipitates formed by seawater neutralization at pH 9.25 for the different Bayer liquor concentrations were also acid digested to determine their elemental composition (**Table** **3**). Magnesium quantities recorded in the lower Bayer liquor concentrations (1–5 g L^−1^) were greater than predicted from the stoichiometry for hydrotalcite material (i.e., 2.70). Therefore, a second magnesium mineralogical phase must be present, which based upon XRD and IR analysis was most probably brucite (Mg(OH)_2_).^23^, ^36^ In addition, the elemental analysis of the precipitates revealed that the Mg:Al mass ratio approached 2.7:1 as the concentration of Bayer liquor increased, which was typical for hydrotalcite.^36^ Calcium in the precipitates (Table 3) was identified by XRD to be calcite and aragonite (Figure 1).

**Table 3:** Concentration of elements in precipitates obtained at pH 9.25 by seawater neutralization of Bayer liquor

Bayer liquor concentration [g L^−1^ Al_2_O_3_]	Mg:Al	Concentration [mg L^−1^]				
		Mg	Al	Ca	Na	S
1	27.29	287.07	10.52	19.67	30.03	10.23
2	13.46	164.63	12.23	22.58	117.67	12.91
3	8.74	169.93	19.44	54.46	72.28	13.17
4	6.21	170.83	27.51	46.98	66.30	11.43
5	5.35	152.46	28.52	42.54	92.61	12.02
6	4.01	181.70	45.27	11.98	69.55	12.33
7	3.94	177.22	44.95	29.21	58.34	26.14
8	3.57	163.97	45.92	38.48	68.20	13.38
9	3.36	165.56	49.34	44.66	63.41	12.99
10	3.08	163.16	52.94	39.58	57.91	13.83

**Figure 4:**
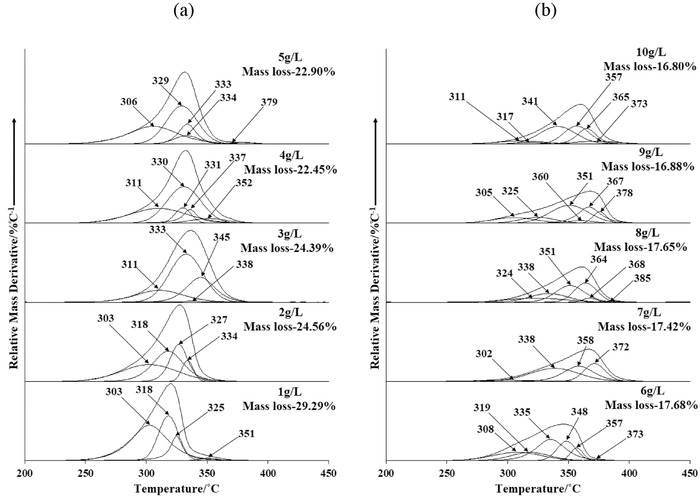
DTG curves of the Bayer precipitates in the dehydroxylation/decarbonation region: a) 1–5 g L^−1^ and b) 6–10 g L^−1^ Bayer liquor.

To provide a deeper insight into the seawater neutralization of Bayer liquor with different alumina compositions, thermal analysis of Bayer precipitates obtained after seawater neutralization of Bayer liquor (1–10 g L^−1^ Al_2_O_3_) at pH 9.25 was completed (**Figure** **4**). Focus was made on the mass loss between 200 and 500 °C associated with the dehydroxylation and decarbonation of hydrotalcite (Equation (5)),^49^, ^50^ dehydroxylation of brucite (Equation (6)),^51^ and dehydroxylation of mixed metal oxides (Equation (7))^49^, ^50^

(5) $$\begin{array}{l} {\mathrm{Mg}}_{6}{\mathrm{Al}}_{2}{\left(\mathrm{OH}\right)}_{16}{\mathrm{CO}}_{3\left(s\right)}\to {\mathrm{MgAl}}_{2}O_{4\left(s\right)}+5{\mathrm{MgO}}_{\left(s\right)}+\;{\mathrm{CO}}_{2\left(g\right)}\\ \;\;\;+6H_{2}O_{\left(l\right)}+2_{2}^{1}O_{2\left(g\right)} \end{array}$$

(6) $$\mathrm{Mg}{\left(\mathrm{OH}\right)}_{2\left(s\right)}\to {\mathrm{MgO}}_{\left(s\right)}+H_{2}O_{\left(g\right)}$$

(7) $${\mathrm{Mg}}_{2}\mathrm{Al}{\left(\mathrm{OH}\right)}_{7\left(s\right)}\to {\mathrm{Mg}}_{2}\mathrm{AlO}{\left(\mathrm{OH}\right)}_{2\left(s\right)}+2H_{2}O_{\left(g\right)}+3\frac{1}{2}O_{2\left(g\right)}+\frac{1}{2}H_{2}$$

According to Palmer and Frost,^50^ weakly bonded interlayer water was removed at between 300 and 330 °C, while the initial dehydroxylation of hydrotalcite layers occurs between 335 and 350 °C. Decomposition processes in the temperature range (360–370 °C) may be due to slight decarbonation of aragonite, whereas the highest temperature region (370–385 °C) typically involves the simultaneous dehydroxylation and decarbonation of hydrotalcite.^50^

It was noted that the decomposition of the hydroxyl layers and interlayer anions undergoes slight changes based on the Bayer liquor concentration (Figure 4). The major decomposition temperature generally increased from 330 to 365 °C as the Bayer liquor concentration increased. Based on the observation of increased water and carbonate anions in the infrared spectra of these precipitates it was postulated that a more structured interlayer was formed, thus explaining the increased thermal stability. In terms of overall mass loss, Bayer precipitates obtained between 1 and 4 g L^−1^ Bayer liquor exhibited a mass loss of 29.29% (1 g L^−1^), 24.56% (2 g L^−1^), 24.39% (3 g L^−1^), and 22.45% (4 g L^−1^). In contrast, mass losses between 17.6 and 16.8% were observed for precipitates obtained from higher concentrations of Bayer liquor (6–10 g L^−1^).

### 2.2 Impact of Bayer Liquor Composition on Neutralization Efficiency

**Figure 5:**
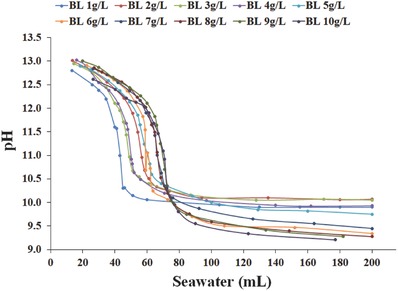
pH variation as a function of seawater addition to Bayer liquor with different alumina concentrations.

The neutralization curves obtained by seawater addition to different Bayer liquor compositions (1–10 g L^−1^ Al_2_O_3_) are shown in **Figure** **5**. As a general observation, three distinct reaction zones were evident: (1) an initial decrease from pH 13 to 12.5; (2) an inflection point between pH 12.5 and 10; and (3) the plateauing of the pH at values less than pH 10. As the Bayer liquor alumina concentration increased, two observations were made: (1) the amount of seawater required to reduce the pH increased, and (2) the final solution pH was lower. This latter observation was believed to be related to the reduced concentration of carbonate for low Bayer liquor concentrations, which in turn resulted in a smaller amount of calcium carbonate being formed (pH buffering agent).

**Figure 6:**
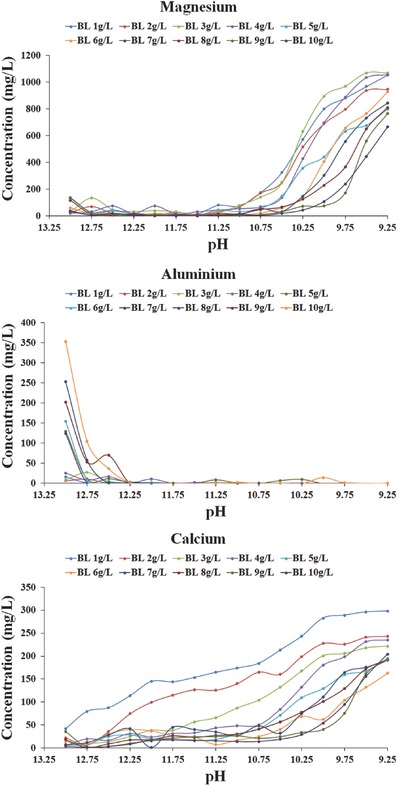
Neutralization curves of different Bayer liquors with varying Al(OH)_4_^−^ concentrations and corresponding Ca^2+^, Mg^2+^, and Al^3+^ in solution.

To gain an insight into the chemistry occurring, which may explain the pH behavior in Figure 5, examination of the concentrations of major ions in solution was made (**Figure** **6**). The initial concentration of magnesium in seawater was 1256 mg L^−1^; however, upon its addition to Bayer liquor the concentration of Mg in the supernatant remained below 100 mg L^−1^ until a pH of ≈11, whereupon the concentration rapidly increased. This enhancement of magnesium concentration in solution corresponded with the inflection points (≈11.5 for 1–5 g L^−1^ Bayer liquor and 10.5 for 6–10 g L^−1^ Bayer liquor) of the neutralization curves.

Consideration of the behavior of aluminum ions in solution provided an insight as to the process occurring. Although the initial concentration of aluminum differed for each Bayer liquor sample, a general trend was that the presence of aluminum in solution decreased until depleted as seawater was added. Notably, the simultaneous removal of aluminum and magnesium from solution at relatively high pH values was consistent with the formation of hydrotalcite during the seawater neutralization process of Bayer liquor.^52^ The XRD patterns of the precipitates confirmed hydrotalcite was present after the neutralization process (Figure 1). However, magnesium was also associated with mineralogical phases such as brucite at low Bayer liquor concentrations (refer to Section 2.1).

The calcium concentration in the supernatant appeared to depend on the Bayer liquor concentration, with deviations in behavior being observed for Bayer liquors 1–4 g L^−1^. At lower Bayer liquor concentrations, a steady rise in solution concentration was observed, which indicated that calcium was not readily consumed in the formation of precipitates. One rationale for this observation was that there was insufficient carbonate in the Bayer liquor to form calcium carbonate. However, at higher Bayer liquor concentrations (5–10 g L^−1^), calcium was consumed as the pH declined to 10.75 before steadily increasing in solution (formation of calcite and/or aragonite).

Therefore, based on previous studies, the driving force behind the reduction of pH of Bayer liquors was due to the formation of hydrotalcite and calcium carbonate species.^36^, ^44^, ^46^, ^52^ It was proposed that at higher Bayer liquor concentrations the increased amount of aluminum (Al(OH)_4_^−^), carbonate (Na_2_CO_3_), and hydroxide (NaOH) present enabled a greater amount of hydrotalcite to form, which resulted in a significant decrease in pH as it formed. For the lower Bayer liquor concentrations (1–5 g L^−1^) tested, the inflection points of the neutralization curve appeared at lesser seawater volumes. This latter observation was believed to be related to the reduced concentration of carbonate in these Bayer liquors, which in turn resulted in a smaller amount of calcium carbonate being formed.

### 2.3 Acid Mine Drainage Treatment with Bayer Precipitates

**Figure 7:**
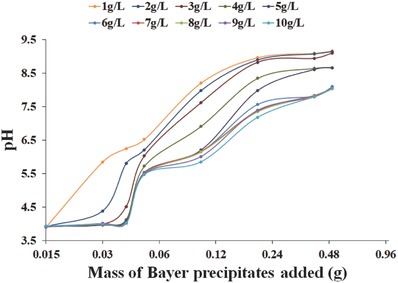
Neutralization curve obtained by the addition of Bayer precipitates to AMD.

The various Bayer precipitates were added to AMD to raise solution pH and remove dissolved species in subsequent precipitates (**Figure** **7**).

The ideal pH range for the discharge of treated AMD solutions into local water bodies should be between 6.0 and 8.5;^53^ all precipitates successfully met this latter condition. Neutralization of AMD water was assumed to be predominately through the release of hydroxyl ions and carbonate species due to the dissolution of brucite (present in 1–3 g L^−1^ Bayer precipitates), mixed metal hydroxide species (hydrotalcite and Mg_2_Al(OH)_7_), and CaCO_3_ species present in all Bayer precipitates (Equations (8)–(11))

(8) $$\mathrm{Mg}{\left(\mathrm{OH}\right)}_{2\left(s\right)}+\;2H_{\left(\mathrm{aq}\right)}^{+}\;\to \;{\mathrm{Mg}}_{\left(\mathrm{aq}\right)}^{2+}+2H_{2}O_{\left(l\right)}$$

(9) $$\begin{array}{l} {\mathrm{Mg}}_{6}{\mathrm{Al}}_{2}{\left(\mathrm{OH}\right)}_{16}{\mathrm{CO}}_{3}\cdot 4H_{2}O_{\left(s\right)}+2H_{\left(\mathrm{aq}\right)}^{+}\to \;6{\mathrm{Mg}}_{\left(\mathrm{aq}\right)}^{2+}\\ \;\;\;+2\mathrm{Al}{\left(\mathrm{OH}\right)}_{3\left(s\right)}+\;{\mathrm{CO}}_{2\left(g\right)}+10H_{2}O_{\left(l\right)} \end{array}$$

(10) $${\mathrm{Mg}}_{2}\mathrm{Al}{\left(\mathrm{OH}\right)}_{7\left(s\right)}+\;4H_{\left(\mathrm{aq}\right)}^{+}\to 2{\mathrm{Mg}}_{\left(\mathrm{aq}\right)}^{2+}+\mathrm{Al}{\left(\mathrm{OH}\right)}_{3\left(s\right)}+\;4H_{2}O_{\left(l\right)}$$

(11) $${\mathrm{CaCO}}_{3\left(s\right)}+\;2H_{\left(\mathrm{aq}\right)}^{+}\;\to {\mathrm{Ca}}_{\left(\mathrm{aq}\right)}^{2+}+{\mathrm{CO}}_{2\left(g\right)}+\;H_{2}O_{\left(l\right)}$$

Gibbsite formed due to the dissolution of hydrotalcite and mixed metal oxides (Equations (9) and (10)), which also has the potential to react with acid and increase the pH as shown in Equation (12)

(12) $$\mathrm{Al}{\left(\mathrm{OH}\right)}_{3\left(s\right)}+\;3H_{\left(\mathrm{aq}\right)}^{+}\;\to {\mathrm{Al}}_{\left(\mathrm{aq}\right)}^{3+}+3H_{2}O_{\left(l\right)}$$

Highest final pH values (9.17–9.11) were achieved by addition of precipitates obtained from lower Bayer liquor concentrations (1–3 g L^−1^), compared to 8.67 (4–5 g L^−1^ Bayer precipitates) and 8.10–8.03 (6–10 g L^−1^ Bayer precipitates). The difference in final solution pH can be described in terms of buffering phenomena. Various authors reported the presence of calcium carbonate species (such as calcite) buffer solution pH, making it difficult to increase the pH > 8.^54^, ^55^, ^56^, ^57^, ^58^ In the case of precipitates obtained from 1 to 3 g L^−1^ Bayer liquor concentrations, relatively high pH was attained due to a lack of carbonate buffering effect. The other reason behind the high pH attained by lower Bayer liquor concentration precipitate was the presence of brucite (Mg(OH)_2_), which has a higher solubility than hydrotalcite.^59^

**Figure 8:**
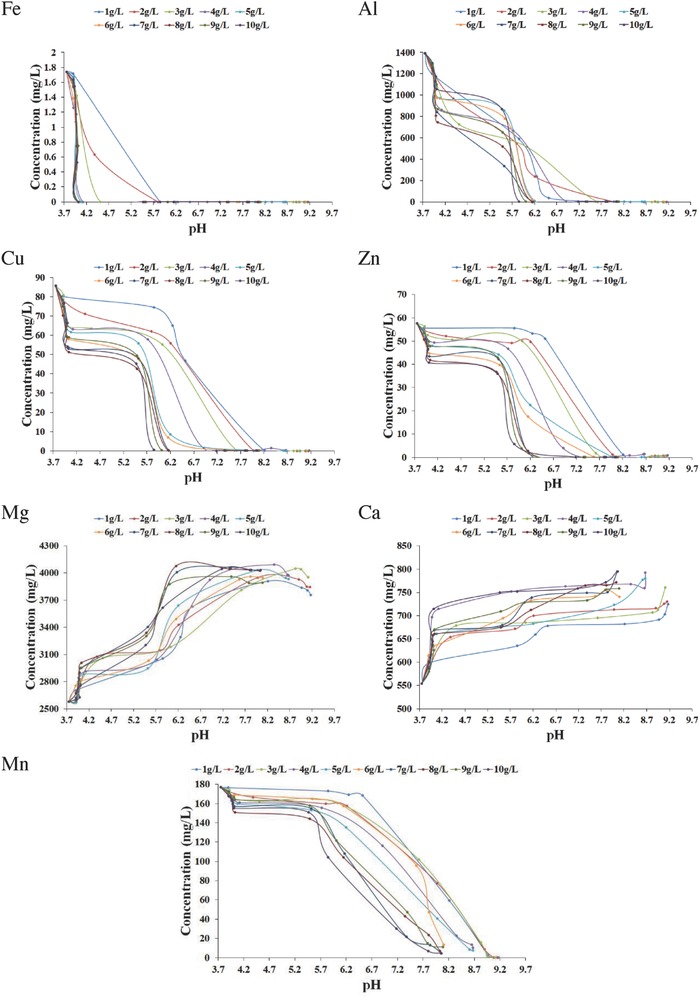
Variation of concentration of dissolved components in AMD as a function of solution pH when Bayer precipitate was added.

To facilitate interpretation of the neutralization curves displayed in Figure 7, the change in concentration of major dissolved species of interest (Al, Mn, Cu, Zn, and Fe) as a function of Bayer precipitate addition was also monitored (**Figure** **8**). In general, the addition of Bayer precipitates reduced the concentrations of all metals to satisfy discharge limits set by the Australian and New Zealand Environment Conservation Council (ANZECC, 2000) guidelines (summarized in a previously published table by the authors).^53^, ^60^ Increasing the pH of AMD water facilitated the precipitation of metals of interest in the following order: Fe, Al, Cu, Zn, and Mn.

Iron appeared to be the easiest metal to remove from solution, with the precipitation of all Fe (below instrument detection limits < 0.05 mg L^−1^) by: pH 5.75 for 1–2 g L^−1^ Bayer precipitates; pH 4.5 for 3–4 g L^−1^ Bayer precipitates; and pH 4.10 for 5–10 g L^−1^ Bayer precipitates (Figure 8). Iron is typically found as ferric (Fe^3+^) ions in AMD waters, due to oxygenation by turbulence.^58^ The precipitation of iron hydroxides at pH > 3.5 has been reported.^61^ Rahman et al. additionally reported the formation of Fe‐Al LDH after the reaction between Fe and Mg‐Al LDH.^59^

The removal of aluminum from AMD water after treatment with 1–10 g L^−1^ Bayer precipitates is also illustrated in Figure 8. For all Bayer precipitates, aluminium precipitation commenced at pH > 4 and its concentration fell below water quality discharge limits at pH ≥ 6.5.^53^, ^60^ At a pH above 5.5, aluminium begins to precipitate as Al(OH)_3_.^62^ The aluminium concentration remained constant below pH 4, thus indicating aluminium did not simultaneously precipitate out with iron. A slight increase in aluminium concentration was observed above pH 8, due to the formation of aluminate ions;^62^ however, the aluminium concentraton remained below the ANZECC (2000) guidelines.^60^

Similarly, it was observed that Cu and Zn concentrations began to decreaese at pH ≥ 4 (Figure 8), thus indicating the possibility that they simultaneously precipitated with aluminium. Complete copper and zinc removal occurred at: pH > 8 for 1–4 g L^−1^ Bayer precipitates; pH > 7.5 for 6–7 g L^−1^ Bayer precipitates; and at pH > 5.9 for 8–10 g L^−1^ Bayer precipitates. Treatment of acidic copper and zinc solutions with Mg‐Al LDH has been postulated to proceed not only via the removal of copper from solution by hydroxide precipitation,^59^, ^63^ but also by isomorphic substitution with magnesium in LDH materials.^64^

The removal of manganese from AMD can be problematic, requiring a pH greater than 9 to produce manganese precipitates, with complete removal achieved at 10.5.^62^, ^65^ However, raising the pH to these levels in AMD waters containing aluminum, results in the dissolution of aluminum hydroxide precipitates. The removal of manganese was recorded in Figure 8 at: pH > 7.5 for 1–3 g L^−1^ Bayer precipitates; at pH > 6.5 for 4–7 g L^−1^ Bayer precipitates; and at pH > 5.8 for 8–10 g L^−1^ Bayer precipitates. Bayer precipitates (1–3 g L^−1^) containing brucite characterised by a pH > 9, were able to achieve complete removal of Mn. Due to the buffering effect of calcium carbonate in 4–10 g L^−1^ Bayer precipitates a maximum pH of 8.5 was achieved, which was insuffecient for the complete removal of Mn.

It was noted that treatment of AMD with 1–10 g L^−1^ Bayer precipitates caused an increase in magnesium and calcium concentrations in the treated water (Figure 8). Increase in calcium concentrations was believed to be due to the dissolution of calcium carbonate species (calcite and aragonite) (Equation (11)), present in precipitates obtained after seawater neutralization of Bayer liquor.^66^ The observed rise in Mg concentration in treated water was ascribed to the dissociation of mixed metal hydroxide species (hydrotalcite and Mg_2_Al(OH)_7_) and brucite ((Mg(OH)_2_) present in Bayer precipitates obtained from 1 to 3 g L^−1^ Bayer liquor.^67^ There is insufficient information available to set acceptable limits for magnesium in livestock drinking water.^68^ For AMD treated with 1–10 g L^−1^ Bayer precipitates, the concentration of calcium after treatment was below the concentration limit set by ANZECC (2000) guidelines (i.e., 1000 mg L^−1^).^60^

## 3 Conclusions

The application of seawater neutralized supernatant liquor waste from the alumina refining industry (Bayer precipitates) has been successfully shown to neutralize AMD water to ANZECC 2000 discharge guidelines. This research has shown that the composition of Bayer precipitates were key to the degree of neutralization and metal removal in AMD waters. Overall, the research provides a deeper understanding of the chemical reactions involved in (1) the dissolution of Bayer precipitates, and (2) the treatment of AMD water using Bayer precipitate dissolution species.

Bayer liquor compositions were found to influence the composition of the precipitates formed. The major materials synthesized were hydrotalcite (Mg_6_Al_2_(OH)_16_CO_3_∙*x*H_2_O), calcite and aragonite (CaCO_3_), mixed metal hydroxides (Mg_2_Al(OH)_7_), and halite (NaCl). Brucite (Mg(OH)_2_) was observed for lower Bayer liquor concentrations (1–3 g L^−1^ Al_2_O_3_) under conditions wherein the concentrations of aluminum and hydroxyl species were unable to produce solely hydrotalcite. The ratio of calcite to aragonite formed was related to the Mg:Ca ratio with calcite dominating at high alumina levels and aragonite relatively more prevalent at low alumina values. Examination of the concentration of individual ions as a function of solution pH (seawater neutralization of Bayer liquor) supported the idea that hydrotalcite was formed in addition to lesser materials such as calcium carbonate and brucite. The degree of buffering capacity, caused by CaCO_3_ species, played a key role in controlling the final pH of solution.

The presence of hydroxide and carbonate species in the hydrotalcite material and the existence of calcium carbonate species in the Bayer precipitates were demonstrated to be amenable for the treatment of acid mine drainage. All Bayer precipitates evaluated, successfully neutralized acid mine drainage. In addition to neutralizing the acidity of AMD, all Bayer precipitates investigated, decreased the concentration of Fe, Al, Cu, and Zn to within acceptable discharge limits (ANZECC, 2000). The removal of Mn to within acceptable limits could only be obtained using the 1–3 g L^−1^ Bayer precipitates due to their ability to attain a higher pH. However, an increase in Al was observed once a pH of 8.5 was obtained; ANZECC (2000) limits for pH were no longer met.

The results of this study showed that Bayer precipitates have the potential to be used as alkali materials in the treatment of AMD waters. Further investigations are required to explore the influence of AMD composition upon the effectiveness of Bayer precipitates and also to determine if the precipitates can be modified to further enhance performance. Particular emphasis should be placed upon a means for removing dissolved manganese species without solubilizing aluminum.

## 4 Experimental Section

*Bayer Liquor Preparation*: Bayer liquor was prepared at a range of concentrations (ranging from 1 to 10 g L^−1^ Al_2_O_3_). **Table** **4** displays information regarding the masses and volumes of chemicals used to prepare these liquors, while the full method can be found in previous work by the authors.^53^ In summary, sodium carbonate (Na_2_CO_3_) was dissolved in deionized (DI) water (1 L) prior to the addition of sodium hydroxide (NaOH) and superevaporative liquor (125.2 g L^−1^ as Al_2_O_3_), before making a final solution volume with DI water (2 L). The SEL was sourced from an Australian Alumina Refinery (location cannot be disclosed due to commercial in confidence). Table 2 provides the elemental compositions of the ten Bayer liquor solutions (1–10 g L^−1^) .

**Table 4:** Masses and volumes of chemicals required to prepare different Bayer liquors

Bayer liquor concentration [g L^−1^ Al_2_O_3_]	Na_2_CO_3_	NaOH	Superevaporative liquor (SEL)
	Mass [g]	Mass [g]	Volume [mL]
1	2.11	67.26	21
2	4.23	63.12	42
3	6.34	58.98	63
4	8.45	54.84	85
5	10.56	50.69	106
6	12.68	46.55	127
7	14.79	42.41	148
8	16.90	38.27	169
9	19.02	34.13	190
10	21.13	29.99	212

*Seawater Neutralization of Bayer Liquor*: Neutralization curves resulting from formation of Bayer precipitates by titrating seawater (sourced from Redcliffe Jetty, Queensland, Australia in 2013; composition provided in Table 2) to Bayer liquor until the desired pH had been reached (increments of 0.25 pH units from pH 13 to 9.5) were collected. This latter procedure involved the slow addition of seawater to Bayer liquor (5 mL), until the desired pH was not only reached but also stabilized after a period of >5 min. Samples were centrifuged (Centurion Scientific C2 Series) after being stirred (24 h, 5 min, 2500 rpm). The supernatant was analyzed using ICP‐OES (syringe filtered, 0.45 μm nylon filter), after dilution using a Hamilton dilutor. The solid was washed (150 mL, DI water) before being centrifuged again, followed by drying overnight in an oven (90 °C).

*AMD Treatment Using Bayer Liquor Precipitates*: The composition of Open Pit AMD water collected at Mount Morgan mine, Queensland, Australia in June 2017 is provided in **Table** **5**. Acid neutralizing capacity was determined by the progressive addition of a quantity (≈0.015–0.5 g) of Bayer precipitates obtained at pH 9.25 from the seawater neutralization of different compositions of Bayer liquors (1–10 g L^−1^ Al_2_O_3_) to acid mine drainage (10 mL). The subsequent mixture was then stirred (24 h) before being centrifuged (Eppendorf Centrifuge 5702, 5 min, 2500 rpm). The supernatant was then transferred into a sample container, while the solid component was washed (10 mL, DI water) before being centrifuged again, and then placed in the oven overnight to dry. A calibrated pH meter (TPS) and probe were used to monitor changes in pH. ICP‐OES was used to monitor elemental changes in solutions.

**Table 5:** Composition of mine pit water from Mount Morgan, June 2017

pH			Conductivity [mS]			SO_4_ [mg L^−1^]		
3.77			14.81			19 342		
Concentration [mg L^−1^]								
Al	Fe	Mn	Cu	Zn	Co	Ni	Cd	Cr
1393	1.74	177	85.77	57.63	4.22	1.19	0.27	0.82
Concentration [mg L^−1^]								
S	Mg	Ca	Na	Si	K	Li	Sr	B
6383	2580	554	632.4	26.87	7.486	0.63	0.93	0.31

*Analysis Methods*: The complete details on the instrumental methods (solid digestion, ICP‐OES, XRD, TGA, and IR spectroscopy) and calibrations used for this study can be found in previous work by the authors.^53^

Solid digestions of Bayer precipitates involved a known mass to be digested in hydrochloric and nitric acid (2:1 ratio), for 1 h at 80 °C, prior to diluting with DI water (final solution volume of 50 mL). Supernatants of the neutralization process and the solid digests were diluted by a Hamilton dilutor prior to analysis using a VISTA‐MPX CCD simultaneous ICP‐OES instrument (calibrated with a certified standard).

Bayer precipitates were characterized by XRD (Philips X'pert wide angle X‐ray diffractometer), TGA (TA Instrument series Q500), and infrared spectroscopy (Nicolet iS50 Fourier transform infrared spectrometer with a diamond ATR (attenuated total reflectance) cell).

## Conflict of Interest

The authors declare no conflict of interest.

## Supporting information

Supplementary
