# Supplementary material for: Acid Mine Drainage Treatment Using Bayer Precipitates Obtained from Seawater Neutralization of Bayer Liquor
Source: Glob Chall. 2018 Sep 21;2(12):1800061. doi: 10.1002/gch2.201800061 (PMC6607373; doi:10.1002/gch2.201800061)
Supplement: Supplementary file 1 — Supplementary [file GCH2-2-1800061-s001.pdf]

Copyright WILEY-VCH Verlag GmbH & Co. KGaA, 69469 Weinheim, Germany, 2018.

Global Challenges Open Access

Supporting Information

for *Global Challenges,* DOI: 10.1002/gch2.201800061

Acid Mine Drainage Treatment Using Bayer Precipitates Obtained from Seawater Neutralization of Bayer Liquor

Gurkiran Kaur, Sara J. Couperthwaite, and Graeme J. Millar*

Supplementary

Table 1: Mass of Bayer precipitate used for acid digestion

Bayer precipitate (g/L)	Mass (g)
1	0.1438
2	0.1446
3	0.0557
4	0.1049
5	0.0853
6	0.1320
7	0.1534
8	0.1371
9	0.1581
10	0.1277

Table 2: Infrared peaks for Bayer precipitates obtained by seawater neutralisation of 1-10 g/L Al_2O_3

Wavenumber range (cm^{-1})	Sample Peaks										Vibrational mode	Assignment
	1 g/L	2 g/L	3 g/L	4 g/L	5 g/L	6 g/L	7 g/L	8 g/L	9 g/L	10 g/L		
3700-3690	3699	3699	-	-	-	-	-	-	-	-	Mg-OH stretching	Brucite
3690-3500	3688<br>3614	3681<br>3641	3689<br>3644	3683<br>3638	3684<br>3640	3685<br>3647	3685<br>3648	3682<br>3646	-<br>3652	3658	Mg-Al-OH stretching	Hydroxyl layer
3500-3300	3527<br>3391	3568<br>3417	3570<br>3411	3562<br>3412	3565<br>3410	3561<br>3423	3561<br>3423	3562<br>3429	3568<br>3439	3572<br>3445	Mg_2Al-OH<br>$MgAl_2$-OH	Hydroxyl layer
3300-3100	3251<br>3190	3246	3234	3238	3226	3289	3270	3269	3285	3308	O-H stretching vibrations of H_2O	H2O coordinated to cations in
3100-2800	-	2992	3018	3074	3003	3008	3014	3064	3000	3085	$H_2O-CO_3^{2-}$ bridging mode	$H_2O-CO_3^{2}$
1660-1600	1639	1639	1641	1638	1639	1635	1636	1637	1634	1634	H_2O bending	Interlayer water, H_2O-
1530-1460	1485	1493	1511<br>1477	1532<br>1486	1528<br>1483	1522<br>1495	1523<br>1486	1484	1519<br>1480	1527<br>1493	ν_3 vibrational mode of CO_3^{2-}	$CaCO_3$ (aragonite)

1460-1400	1428	1434	1443<br>1424	1443<br>1420	1443<br>1416	1458<br>1434	1445<br>1407	-<br>1425	1423	1457<br>1414	ν_3 vibrational mode of CO_3^{2-}	$CaCO_3$ (calcite)
1400-1350	-	-	1358	1368	1379	1385	1362	1375	1389<br>1365	1373	Antisymmetric stretch of	H_2O-CO_3^{2-} in
1180-1080	1173<br>1133<br>1097	1175<br>1125<br>1075	1179<br>1129<br>1082	1179<br>1128<br>1081	1170<br>1126<br>1109	1180<br>1145<br>1114	1175<br>1108	1175<br>1135<br>1108	1174<br>1135<br>1109	1172<br>1137<br>1108	ν_1 vibrational mode of CO_3^{2-}	$CaCO_3$
